# Whole Exome Sequencing Reveals Novel and Recurrent Disease-Causing Variants in Lens Specific Gap Junctional Protein Encoding Genes Causing Congenital Cataract

**DOI:** 10.3390/genes11050512

**Published:** 2020-05-06

**Authors:** Vanita Berry, Alex Ionides, Nikolas Pontikos, Ismail Moghul, Anthony T. Moore, Roy A. Quinlan, Michel Michaelides

**Affiliations:** 1 UCL Institute of Ophthalmology, University College London, 11-43 Bath Street, London EC1V 9EL, UK; n.pontikos@ucl.ac.uk; 2 Moorfields Eye Hospital NHS Foundation Trust, London EC1V 2PD, UK; alex.Ionides@moorfields.nhs.uk (A.I.); tony.moore@ucsf.edu (A.T.M.); 3 UCL Cancer Institute, University College London, London WC1E 6BT, UK; ucbtmog@ucl.ac.uk; 4 Ophthalmology Department, University of California School of Medicine, San Francisco, CA 94158, USA; 5 Department of Biosciences, University of Durham, Upper Mountjoy Science Site, Durham DH1 3LE, UK; r.a.quinlan@durham.ac.uk

**Keywords:** whole exome sequencing, autosomal dominant congenital cataract, *GJA3*, *GJA8*

## Abstract

Pediatric cataract is clinically and genetically heterogeneous and is the most common cause of childhood blindness worldwide. In this study, we aimed to identify disease-causing variants in three large British families and one isolated case with autosomal dominant congenital cataract, using whole exome sequencing. We identified four different heterozygous variants, three in the large families and one in the isolated case. Family A, with a novel missense variant (c.178G>C, p.Gly60Arg) in *GJA8* with lamellar cataract; family B, with a recurrent variant in *GJA8* (c.262C>T, p.Pro88Ser) associated with nuclear cataract; and family C, with a novel variant in *GJA3* (c.771dupC, p.Ser258GlnfsTer68) causing a lamellar phenotype. Individual D had a novel variant in *GJA3* (c.82G>T, p.Val28Leu) associated with congenital cataract. Each sequence variant was found to co-segregate with disease. Here, we report three novel and one recurrent disease-causing sequence variant in the gap junctional protein encoding genes causing autosomal dominant congenital cataract. Our study further extends the mutation spectrum of these genes and further facilitates clinical diagnosis. A recurrent p.P88S variant in *GJA8* causing isolated nuclear cataract provides evidence of further phenotypic heterogeneity associated with this variant.

## 1. Introduction

Inherited cataract is a phenotypically and genotypically heterogeneous condition causing visual impairment either from birth or in early infancy. The WHO has estimated that >14 million children are bilaterally blind from cataract, representing >50% of all causes of pediatric blindness globally [1]. Congenital cataracts (CC) are present in 1–6/10,000 live births in developed countries and 5–15/10,000 live births in developing countries. Most vision loss associated with CC is due to amblyopia, but some is due to postoperative complications such as glaucoma and retinal detachment [2]. Congenital cataract can be inherited as an isolated condition, or as a part of other systemic disorders including other ocular defects. Autosomal dominant (AD) is the most common mode of inheritance, followed by autosomal recessive and X-linked recessive. Inherited cataracts are clinically highly heterogeneous, and the phenotype broadly reflects spatiotemporal insults experienced by the developing lens, such as: nuclear, cortical, complete, blue-dot, anterior polar, posterior polar, pulverulent, lamellar, coralliform, posterior nuclear and polymorphic [3,4,5,6].

So far, over 38 disease-causing genes have been identified as being associated primarily with isolated cataract. Pathogenic variants have been identified in genes encoding many different proteins, including intracellular lens proteins (α-, β-, and γ-crystallins), water channel proteins (aquaporins), cytoskeletal proteins (including *BFSP1* [filensin], *BFSP2* [phakinin] and *VIM* [vimentin]), transcription factors (including *FOXE3*, *PAX6*, *PITX3* and *MAFA*), genes with various functions (*EPHA2*, *FYCO1*, *TDRD7*), and membrane gap junction proteins (*GJA3* and *GJA8*, also known as Connexin 46 and Connexin 50, respectively) [7,8].

Connexins are important in cell-to-cell communication, controlling cell growth, cell differentiation and maintaining lens cell homeostasis. During lens development, this communication is maintained via gap junction channels, which permits the flow of ions, metabolites and second messengers between lens fiber cells. In the lens, these channels are made up of three connexin isoforms: *GJA1* (Cx43), *GJA3* (Cx46) and *GJA8* (Cx50). Six connexin molecules, resulting from combinations of the three isoforms, assemble to form a hemichannel or connexon. These hemichannels dock with a counterpart in an adjacent cell to make a gap junction channel linked by their extracellular loops [9,10]. To date, fifty-six heterozygous variants and one homozygous variant have been found in *GJA3*, with various associated lens phenotypes, including pulverulent, nuclear, lamellar, coralliform and total. Ninety-one heterozygous variants in *GJA8* have been described in families with autosomal dominant cataract, and a single homozygous variant in autosomal recessive cataract has been associated not only with inherited cataract, but also age-related cataract and other eye anomalies including microcornea, microphthalmia and corneal opacification [11].

Here, we have undertaken whole exome sequencing (WES), in order to identify variants underlying autosomal dominant congenital cataract (ADCC) in three large families of British origin and an isolated individual with congenital cataract.

## 2. Materials and Methods

### 2.1. Phenotyping

In this study, all the three families were identified through the proband attending the genetic service at Moorfields Eye Hospital and were approved by the local ethics committee of genetic service at Moorfields Eye Hospital, London, UK. All individuals taking part in this research gave written informed consent. All of the family members underwent full ophthalmic examination, including slit lamp examination; all affected individuals were diagnosed as having isolated congenital cataract.

### 2.2. Whole Exome Sequencing (WES) and Bioinformatics Analysis

Genomic DNA was extracted from EDTA sequestered blood samples using the Nucleon II DNA Extraction Kit (Scotlab Bioscience, Strathclyde, Scotland, UK). The DNA samples were sequenced at Macrogen Europe. Exon capture and target enrichment was performed using the SureSelectXT Human All Exon V6 post, (Agilent, Santa Rosa, CA, USA). Paired-end sequencing was performed on an Illumina Hiseq 2500 high-throughput sequencer, generating mean exome coverage of 50×. Raw data in FASTQ format were analysed using the Phenopolis bioinformatics platform [12]. The short-read sequence data were aligned using NovaAlign (version 3.02.08). Variants and indels were called according to GATK (version 3.5.0) best practices (joint variant calling followed by variant quality score recalibration). The variants were then annotated using the Variant Effect Predictor (VEP) [13]. Variants with a sequencing depth of less than 20× were filtered out. Variants were then filtered to only contain novel (not present in public control databases Kaviar [14] and gnomADv2.1.1 or rare variants (gnomAD allele frequency less than 0.0001), in nearly 356 known cataract genes (https://cat-map.wustl.edu/) and predicted to be moderately or highly damaging (CADD>15). Filtered variants were sorted by decreasing the CADD score.

### 2.3. Sanger Sequencing

Bi-directional direct Sanger sequencing was performed to validate the variant identified by next-generation sequencing. Genomic DNA was amplified by PCR using GoTaq 2× Master Mix (AB gene; Thermo Scientific, Epsom, UK) and GJA8 and GJA3-specific primers designed with Primer3.

PCR conditions were as follows: 94 °C for 5 min of initial denaturation, followed by 30 cycles of amplification of 30 s at 94 °C, 30 s at 60 °C, and 45 s at 72 °C. After the PCR products were reacted with BigDye Terminator v3.1, they were run on an ABI 3730 Genetic Analyzer (both from Applied Biosystems, Foster City, CA, USA) and analysed using SeqMan Pro (version 8.0.2 from DNASTAR) sequence analysis. After validating the variant, family segregation was performed in all the individuals.

The protein structure of GJA8 was analysed using SWISSMODEL. (https://swissmodel.expasy.org/repository/uniprot/P48165).

## 3. Results

### Cataract Families

In this study, we have investigated three families with congenital cataract, family A, B and C, and in an isolated individual D.

**Figure 1:**
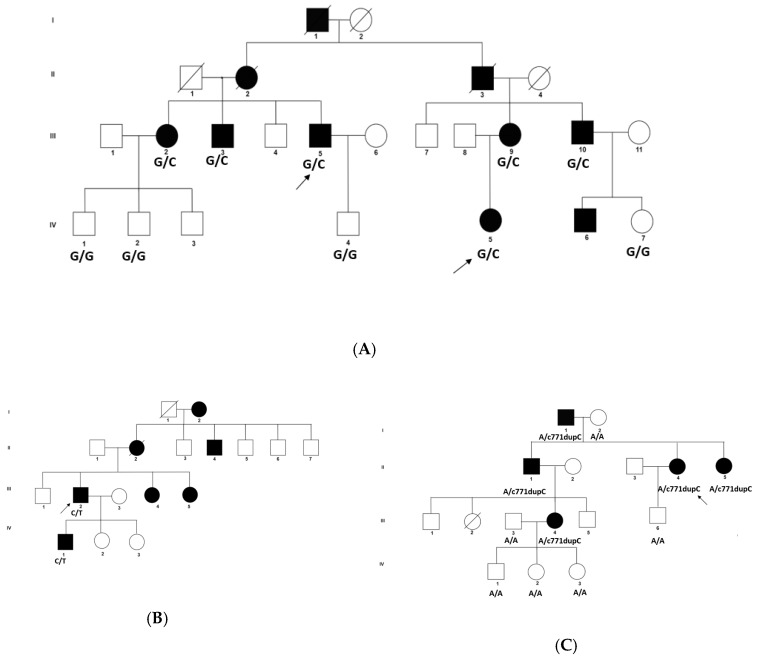
(**A**) Family A: Abridged pedigree with lamellar cataract; (**B**) Family B: Abridged pedigree with nuclear cataract; (**C**) Family C: Abridged pedigree with lamellar cataract. Squares and circles symbolise males and females, respectively. Open and filled symbols indicate unaffected and affected individuals, respectively. The arrow indicates the family members who participated in the whole exome sequencing (WES) analysis. All the members available in the family were sequenced to show the segregation.

**Figure 2:**
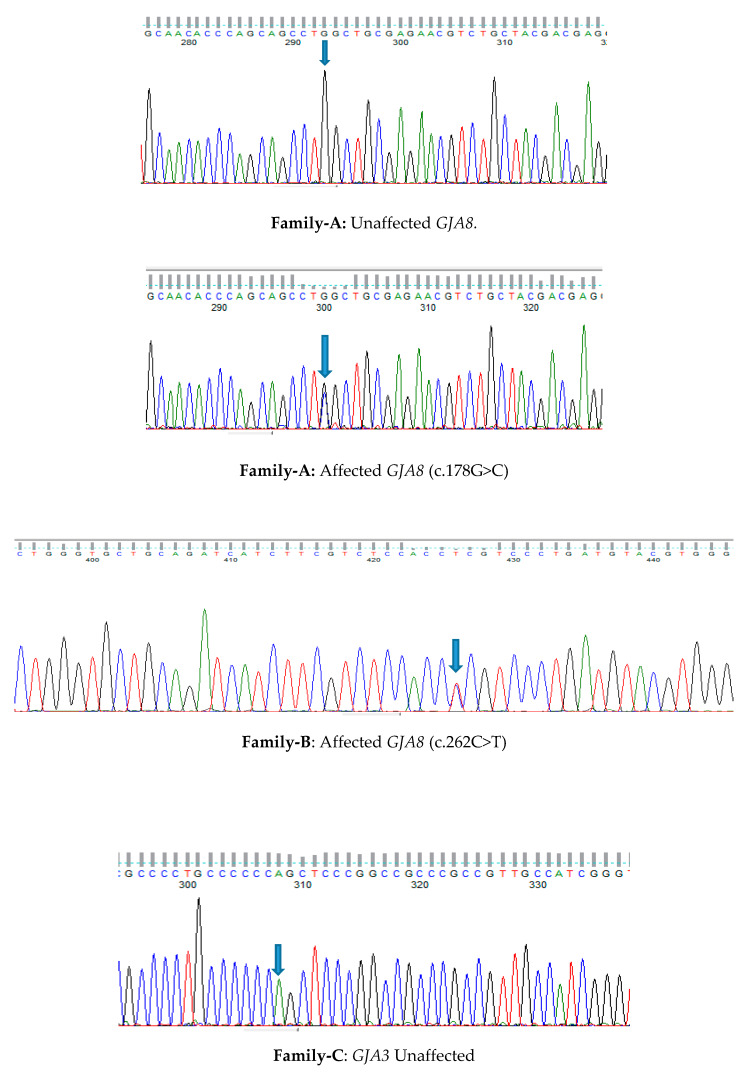

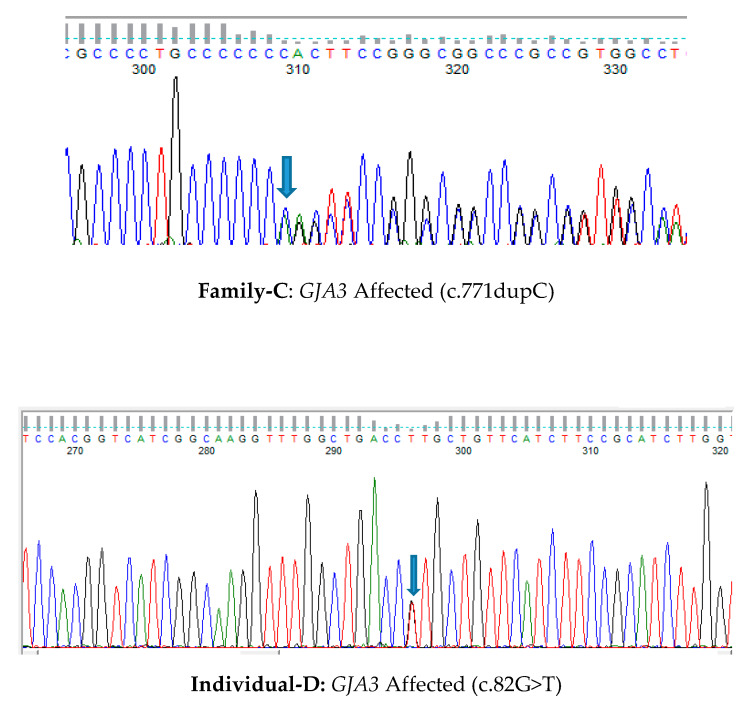
Sequence analysis of *GJA8*—the missense variant c.178G>C shown in an affected member of the family (**A**) with lamellar cataract; sequence analysis of *GJA8*—the missense variant c.262C>T (P88S) shown in an affected member of the family (**B**) with nuclear cataract; sequence analysis of *GJA3*—the missense variant c.771dupC, S258Qfs*68 (p.ser258GlnfsTer68 shown in an affected member of the family (**C**) with lamellar cataract, (**D**) sequence analysis of *GJA3*—the missense variant at c.82G>T in an affected individual with congenital cataract.

**Figure 3:**
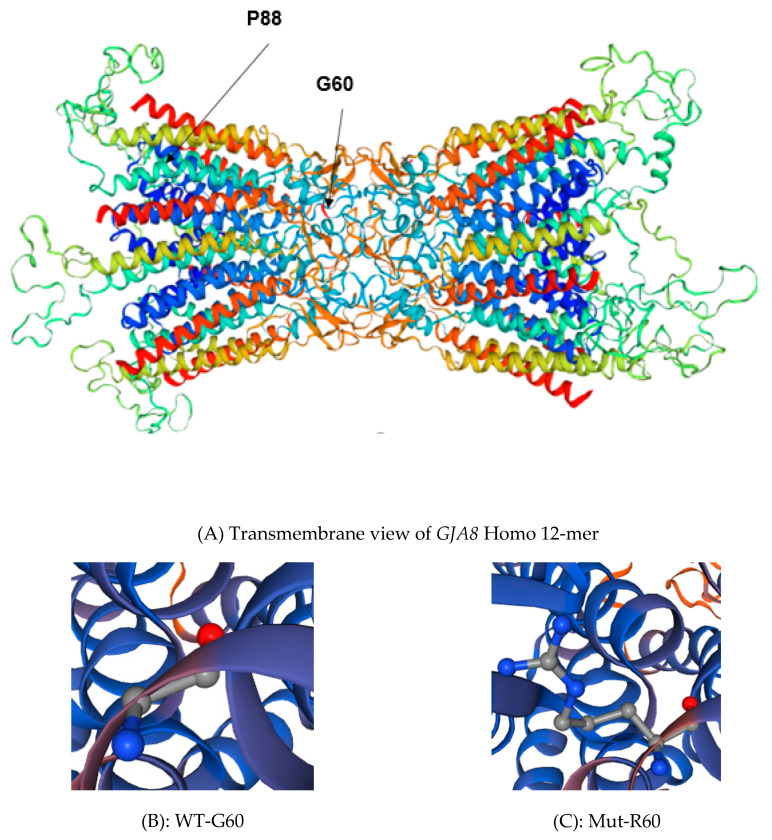
(**A**) Transmembrane view of *GJA8*: (https://swissmodel.expasy.org/repository/uniprot/P48165). (**B**) Wild-type amino at position 60 (Glycine); (**C**) Mutant amino acid at position 60 (Arginine). The side chain of the arginine interferes with the hemichannel activity in the highly conserved region as shown on the protein structure of GJA8 (Figure 3).

Family A is a four-generation pedigree of 18 individuals, with 7 affected, 7 unaffected, and 4 spouses. All individuals were examined, and all affected members had evidence of lamellar cataract and had surgery in infancy (Figure 1A). WES was undertaken in two affected individuals (III-5, IV-5). Variant annotation and filtering were performed using the Phenopolis platform. After filtering, from a total 128,867 variants of 25 variants were filtered in the family, two of which were found to be co-segregated in the two affected individuals. The variant with the highest CADD score was a rare heterozygous damaging variant, NM_005267.5: c.178G>C, p.G60R in the *GJA8* gene on chromosome1q21.1. Direct sequencing confirmed that the missense variant c.178G>C in exon 2 of *GJA8* cosegregated with all the affected members of the family (Figure 2A). This single base change is predicted to result in a glycine (G) to arginine (R) amino-acid substitution (p.G60R) in the first extracellular loop of GJA8 protein (Figure 3A–C).

**Figure 4:**
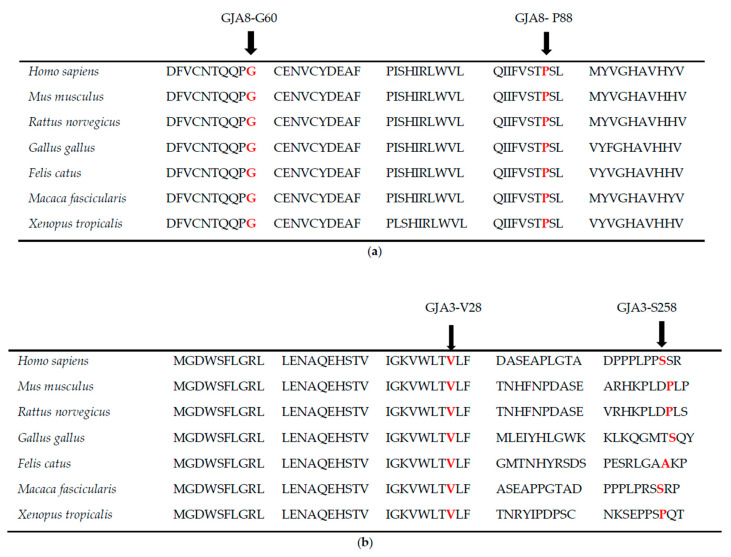
(**a**): The multiple-sequence alignments from different vertebrate species. Arrows show conserved glycine at p.60 and proline at p88. (https://www.ncbi.nlm.nih.gov/nuccore/?term=GJA8). (**b**): The multiple-sequence alignments from different vertebrate species. Arrows show conserved valine at p.28 and serine at p258. (https://www.ncbi.nlm.nih.gov/nuccore/?term=GJA3).

Family B is a four-generation pedigree, with six affected, seven unaffected, and two spouses; all were examined, and all affected individuals had nuclear cataract (Figure 1B). WES was undertaken in one affected individual (III-2). Variant annotation and filtering were performed using the Phenopolis platform. After the Phenopolis genetic variant analysis pipeline, variants were filtered by allele frequency and from a total of 101,893 variants, 5 variants remained. The top scoring variant for CADD was a rare heterozygous variant NM_005267.5 c.262C>T; p.P88S in exon 2 of *GJA8* with a score of 27.60. Direct sequencing confirmed the variant (Figure 2B), which cosegregated in all the affected members of the family (Figure 2B). The p.P88S substitution is located in the second transmembrane of the GJA8 protein. Both of these amino acids p.60 and p.88 are conserved among various species (Figure 4A).

Family C is a four-generation pedigree of 15 members, including 5 affected, 6 unaffected, and 4 spouses. All of the family members were examined, and an isolated lamellar cataract was seen in all affected members. WES was undertaken in one affected individual (II-4) (Figure 1C). After the Phenopolis genetic variant analysis and filtering, from a total of 104,972 variants four remained in individual II-4. The two top scoring variants for CADD were FIGN: p. Thr473Ile with a CADD score of 24.30 and a rare indel variant NM_021954.4 c.771dupC p. S258Qfs*68 in exon 2 of *GJA3* on chromosome 13q11-q12 with a CADD score of 19.38. Direct sequencing confirmed the variant (Figure 2C), which cosegregated in all of the affected members of the family (Family 1C). The Ser258Glnfs is located in the cytoplasmic C-terminal end of the GJA3 protein (Figure 4B).

Individual D with congenital cataract was sent for WES. Following variant analysis and filtering using Phenopolis, from 99711 variants, three variants remained, two variants remained. The top scoring variant (CADD of 32.00) was a novel mutation pVal28Leu in *GJA3* (Figure 2D). Both of these amino acids p.28 and p.258 are conserved among various species (Figure 4B).

All of the four connexin variants are shown in Table 1.

## 4. Discussion

The crystallin lens is an avascular and ever-growing organ in the body, composed of one cell type, lens epithelial cells (LEC), which differentiate into lens fibers (LF) at the equators of the lens. Therefore, to maintain its life-long transparency and nourishment, especially in the mature fiber cells of the lens core, it has developed a specialised intercellular communication mechanism via connexins [8,9]. Connexins (Cx) constitute a large family of transmembrane proteins including 20 members, expressed in various tissues, with 11 out of 20 connexin members having been implicated in many diseases.

Disease-causing variants in Cx26, Cx30 and Cx31 have been associated with deafness; Cx32 with X-linked Charcot–Marie–Tooth disease (CMTX); Cx26 and Cx31 in familial skin disorders and Cx43 in cardiovascular abnormalities [15,16,17]. In the lens, variants in Cx46 and Cx50 are responsible for causing cataract. A Cx50-deficient mouse has also been shown to develop cataract [18]. Connexins share the same membrane topology amongst all of the family members, comprised of two exons encoding a transmembrane protein of 435 amino acids in GJA3 and 433 amino acids in GJA8 respectively, containing four transmembrane domains, two extracellular loops, an intracellular loop, and cytoplasmic amino and carboxy termini [19].

In the lens, three connexins (43, 46 and 50) are expressed during lens development, and thereafter. Cx43 is only expressed in the LEC during the early stages of lens development, but is not associated with lens pathology [20,21]. Cx46 is expressed in the lens epithelial cells and in the lens fibers along with Cx50. Connexin variants constitute approximately 22% of all of the non-syndromic familial cataracts, and are the second commonest cause of non-syndromic cataract after the crystallins. Recently, Myers et al. studied the structure of lens gap junctional channels made by Cx50 and Cx46, and their coassembly in the neighboring cells using cryo-electron microscopy. Their work explains how the hot-spots of disease-causing variants map to the core structural-functional elements, which are linked to hereditary lens opacities [22].

Here, we report four heterozygous variants, three families and one isolated individual of British origin with ADCC. Families A and B harbour two different variants in Cx50. In family A, the novel heterozygous variant at c.178G>C responsible for an AD congenital lamellar cataract resulted in a glycine (nonpolar-hydrophobic) to arginine (positively charged) substitution at position 60 (G60R) of Cx50. The Gly-60 residue of the GJA8 protein within the first extracellular loop of all vertebrate connexins is phylogenetically highly conserved (Figure 4a). This suggests that Gly60 is likely to be functionally important, and therefore, mutation to a basic residue (Arg60) will not only introduce structural constraints, but also change the local ionic environment that we expect to have an adverse effect on protein function. Several studies have shown that the N-terminal (NT) domain along with the first extracellular loop (EL1) and transmembrane 1 (TM1) contribute to the pore lining region of the hemichannel, and therefore sequence changes can potentially interfere with conformation and voltage gating [23,24,25,26,27]. Thus, the G60R substitution may induce a defect in the EL1, leading to conformational changes in the protein to impair the Cx50-mediated coupling of lens fiber cells, hence causing CC.

A recurrent p.Pro88Ser substitution found in family B in Cx50 responsible for autosomal dominant congenital nuclear cataract, resides in the second transmembrane domain of the protein. The p.Pro88Ser variant has previously been reported to cause an autosomal dominant congenital zonular pulverulent cataract in humans [28]. The recurrent Pro88Ser variant thereby displays phenotypic heterogeneity, perhaps due to the role of genetic modifiers acting during the early stages of lens development [29]. Two other autosomal dominant cataracts causing variants, p.Pro88Gln [30] and p.Pro88Thr [31], have previously been localised to the second transmembrane domain. Functional studies of these have shown that both substitutions compromised Cx50 targeting to the plasma membrane, with p.Pro88Gln accumulating in the endoplasmic-reticulum (ER)-Golgi-complex, and p.Pro88Thr forming discrete cytoplasmic inclusions [32].

The novel p.Ser258GlnfsTer68 frameshift variant identified in family C resulted from a cytosine insertion that introduced a premature translation stop codon, located in the cytoplasmic carboxy-terminal region of Cx46 protein. Four other disease-causing variants, c.1137dupC, c.1189dupG, c.1200dupC, c.1152insG, in GJA3 have previously been localised to the same region of the protein with all causing cataract [33,34,35,36]. Several missense or truncated carboxy-terminal Cx32 mutants linked to CMTX have also been shown to cause loss or reduction in function [37,38]. This difference may be explained by decreased translation and/or enhanced degradation [16]. Individual D harboured a novel variant (c.82G>T resulted in Val28Leu in the first transmembrane, a highly conserved region of GJA3. Previously, Devi et al. reported a p.Val28Met pathogenic variant causing a variable cataract phenotype in a family of Indian origin [39]. It suggests that steric hindrance and polarity as a result of a valine substitution indeed affect channel function. Methionine is an amphipathic amino acid whilst valine is hydrophobic and is the most common valine substitution to cause disease in protein transmembrane domains [40].

Our study further extends the mutation spectrum of these two connexin genes and further facilitates clinical diagnosis. The recurrent P88S variant in GJA8 causing isolated nuclear cataract provides evidence of further phenotypic heterogeneity associated with this variant, and highlights the importance of combining clinical observation with WES in order to understand the biological basis for phenotypic variation associated with inherited cataract [41] as a valuable paradigm to understand the genetic basis of human disease.

## Figures and Tables

**Table 1:** Connexin variants implicated in autosomal dominant congenital cataract (ADCC) families.

Family	Variant	Gene	HGVSc	HGVSp	Phenotype	CADD	GERP	Mutation Taster/Verdict
**A**	*chr1-147380260-G-C*	*GJA8*	c.178G>C	G60R	Lamellar	27.10	5.1999	Disease causing-0.81/ Likely Pathogenic
**B**	*chr1-147380344-C-T*	*GJA8*	c.262C>T	P88S	Nuclear	27.60	5.1999	Disease causing-0.81/Pathogenic
**C**	*chr13-20716657--G*	*GJA3*	c.771dupC	S258Qfs	Lamellar	19.38	4.1749	Likely Pathogenic
**D**	*chr13-20717346-C-A*	*GJA3*	c.82G>T	V28L	Congenital Cataract	32	5.5999	Likely Pathogenic
